# Educating educators on research on research

**DOI:** 10.1007/s40037-021-00662-z

**Published:** 2021-04-20

**Authors:** John P. A. Ioannidis

**Affiliations:** Departments of Medicine, of Epidemiology and Population Health, of Biomedical Data Science, and of Statistics, and Meta-Research Innovation Center at Stanford (METRICS), Stanford University, Stanford, CA USA

Medical education combines science and art. To the extent that it is an art, it depends on charisma. We cannot precisely define charisma. Maybe the common characteristic of charismatic medical educators is that they lack common characteristics. They are all exceptional people possessing idiosyncratic wisdom, some special signature of dealing with patients, or the magic of inspiring mentees [1]. Their teaching is also difficult to put into ISO types of standards. These people simply stand out, which also means (unfortunately) they are rare. Much medical education is not permeated with charisma. Conversely, it can be massively pedestrian: uninspired, uninformed, and suspiciously useless.

By some enforced necessity millions of physicians need to take billions of credit points in courses, complete numerous rotations, and pass scores of certifying exams. They are also exposed to billions of patient encounters during which they may learn something—or get taught something wrong. Medical education is continuous and lifelong. It is never too late to learn something—or to be taught something wrong. Finally, medical educators include both people who define themselves as educators (e.g., “professor” or “residency director”) and others who may be even more influential educators, yet they don’t even know it (e.g., patients).

Any efforts to infuse some rigorous science into the chaotic maze and life experience of medical education is welcome. Since major charisma is so rare, most medical educators would be better off if they have good science to back how, when, where, and with what methods their efforts to educate others and themselves might be most fruitful. A vast research literature has thus sprung up trying to fill this role.

Medical educators report valuing rigorous, feasible, relevant, and applicable research [2]. However, most of their literature does not meet these standards [3]. Most papers present retrospective, uncontrolled data on single institutions and with suboptimal statistical methods [3]. These may still have some descriptive value, e.g., documenting a snapshot experience, but generalizability is low and validity questionable. Unfortunately, little funding is given to medical education research, and this limits the ambition of much work done in the field. Unsurpringly, funding is associated with higher quality medical education research [4].

Even when interventions are assessed, often, outcomes of these studies are not what one would wish to see. Major outcomes (e.g., impact of patient outcomes and career-long satisfaction or burn-out of learners) are difficult to investigate. There is even confusion on what are the best outcomes [5]. As it stands, short-term, easy-to-capture surrogates predominate.

Experimental studies with rigorous randomized controls are a minority, even though experimental work has become more common across all social sciences, including education [6]. However, experimental studies often depend more on short-term surrogates. The perfect study, with informative placement against any pre-existing evidence, unbiased experimental controls, long-term follow-up, and highly relevant, pragmatic outcomes may not exist. Systematic reviews have become very popular and occasionally they may offer more insights, but even for them much is desired in terms of validity, utility, and readiness to apply the evidence [7].

Given the vast amount of published research and the ingrained problems of this research, doing research on research has become increasingly attractive in many scientific fields [8]. Medical education cannot escape this pattern. It is not good enough to just realize that much of the scientific literature is not very reliable and the vast majority (even among what is relatively reliable) is useless. Going further, many scientific practices are also rapidly being transformed, with new frontiers opened by enhanced analytical capability, new statistical methods, new standards of peer review and dissemination of scientific information, enhanced openness, an intense soul-searching on methods to appraise evidence, questioning of the current methods to appraise and reward scientists—and more.

Medical educators need to be cognizant of this ongoing revolution. Some may even lead the revolution, doing work on pioneering new, better methods, or research on research that identifies and disseminates better methods. The vast majority of medical educators may not do research themselves, but would still benefit by being trained to use the best research and what we learn from that best research.

We need to educate the educators not only on research, but also on research on research. How to best do this is perhaps yet another research question, but it should have firm foundations in real-life considerations. We cannot expect every medical educator to become an investigator. In fact, past incentive structures that forced physicians to write research papers (a rather weird prerequisite for many purely clinical jobs), have backfired [9]. These incentives may have contributed to the biomedical literature waste produced by unqualified researchers. Systematic review shows that incentivizing scholarly work during residency increases the number of publications and presentations residents produced [10]. However, it is quality, rather than just productivity, that should matter. Most medical educators do not need to pursue research. Nevertheless, they need to be educated to have the skills so as to be helped by research—or at least not be fooled by it.

Requesting comprehensive training on research methods is impossible and impractical. It typically ends in spurious subpar training, e.g., statistics-lite education leading to major misconceptions that are propagated both in research and practice. Medical educators may best be educated on higher-level, bird’s-eye view concepts, and thus be able to evaluate and use research findings that might positively impact their teaching practices, their medical practices, or both, based on the soundest evidence. In this process, understanding research on research principles may be helpful. These principles may offer foundational knowledge on the scientific method and on how evidence can be calibrated.

In the end, we cannot educate educators on every little subject matter item they should know; this would take more than a lifetime. Perhaps, we can educate them on how to calibrate their expectations about how they might get to know something—and how often they need to acknowledge that we simply don’t know. But then, it also takes some charisma (just a bit, not much) to acknowledge what we don’t know and remain open to learning.
